# Depression literacy of undergraduates in a non-western developing context: the case of Sri Lanka

**DOI:** 10.1186/s13104-015-1589-7

**Published:** 2015-10-22

**Authors:** Santushi D. Amarasuriya, Anthony F. Jorm, Nicola J. Reavley

**Affiliations:** Behavioural Sciences Stream, Faculty of Medicine, University of Colombo, 25, Kynsey Road, PO Box 271, Colombo 8, Sri Lanka; Centre for Mental Health, Melbourne School of Population and Global Health, University of Melbourne, 207 Bouverie Street, Parkville, VIC 3010 Australia

**Keywords:** Depression literacy, Mental health literacy, Help-seeking, Beliefs, Intentions, Recognition, Labelling, Undergraduate, Depression, Cultural differences

## Abstract

**Background:**

Research examining the depression literacy of undergraduates in non-western developing countries is limited. This study explores this among undergraduates in Sri Lanka.

**Methods:**

A total of 4671 undergraduates responded to a survey presenting a vignette of a depressed undergraduate. They were asked to identify the problem, describe their intended help-seeking actions if affected by it and rate the helpfulness of a range of help-providers and interventions for dealing with it. Mental health experts also rated these options, providing a benchmark for assessing the undergraduates’ responses.

**Results:**

Only 17.4 % of undergraduates recognised depression, but this was significantly lower among those responding in Sinhala compared to English (3.5 vs 36.8 %). More undergraduates indicated intentions of seeking informal help, such as from friends and parents, than from professionals, such as psychiatrists and counsellors. However, a majority rated all these help-providers as ‘helpful’, aligning with expert opinion. Other options recommended by experts and rated as ‘helpful’ by a large proportion of undergraduates included counselling/psychological therapy and self-help strategies such as doing enjoyable activities and meditation/yoga/relaxation exercises. However, a low proportion of undergraduates rated “western medicine to improve mood” as ‘helpful’, deviating from expert opinion. Although not endorsed by experts, undergraduates indicated intentions of using religious strategies, highly endorsing these as ‘helpful’. Labelling the problem as *depression* and using mental health-related labels were both associated with higher odds of endorsing professional help, with the label ‘depression’ associated with endorsing a wider range of professional options.

**Conclusions:**

The recognition rate of depression might be associated with the language used to label it. These undergraduates’ knowledge about the use of medication for depression needs improvement. Health promotion interventions for depressed undergraduates must be designed in light of the prevalent socio-cultural backdrop, such as the undergraduates’ high endorsement of informal and culturally relevant help-seeking. Improving their ability to recognise the problem as being mental health-related might trigger their use of professional options of help.

**Electronic supplementary material:**

The online version of this article (doi:10.1186/s13104-015-1589-7) contains supplementary material, which is available to authorized users.

## Background

The high prevalence of depression among undergraduates is a matter needing immediate attention [1, 2]. Not only do undergraduates report a lack of need [3] and low level of intention [4] to seek help for depression, a majority of severely distressed undergraduates, many who might be depressed, do not seek help for their problems [5–7]. Among factors affecting a young person’s help-seeking is their ability to recognise a condition [8] and their knowledge about mental health issues and sources of help [9], considered within the realm of mental health literacy.

Jorm et al. [10] describe mental health literacy as “knowledge and beliefs about mental disorders which aid their recognition, management or prevention” (p 182). Although there is a large body of research examining mental health literacy of various populations, with depression extensively studied, most research focuses on developed countries, with inadequate examination of this area in the non-western developing world [11, 12]. Hence, this study focuses on examining depression literacy of undergraduates in Sri Lanka in the areas of problem-recognition and knowledge about dealing with depression.

### The case of Sri Lanka

Although there have been prior examinations of mental health literacy in Sri Lanka, one has been specifically on personal carers of patients with depression and schizophrenia [13] and the other on the general public and professionals, where a considerable proportion of participants were health professionals or teachers [14]. There has been no comprehensive study examining mental health literacy or mental-health-related practices of undergraduates in Sri Lanka thus far, indicating, the need for the present study. Furthermore, given that mental health professionals in the country are limited [15] it becomes necessary to identify undergraduates’ knowledge and responses relating to mental disorders to develop feasible and effective mental health responses suitable for them.

However, the examination of depression literacy in this cultural context poses several challenges. An examination of the population’s ability to recognise depression can be complicated by the lack of an equivalent lay term for depression in Sinhalese, the language spoken by a majority of the population, with words used for the condition, such as *visaadaya* or *maanasika avapeedanaya*, being those devised by clinicians [16, 17]. However, evidence that almost 10 % of undergraduates in Sri Lanka screen positive for Major Depression [18] emphasises the need for examining whether they recognise the condition and how they label it. Furthermore, evidence that young persons and undergraduates who recognise depression show better treatment preferences and actions, such as getting help from professionals [19, 20], highlight the need for examining whether this undergraduate population’s ability to recognise depression and the labels they use are associated with their help-seeking for the problem.

Another aspect to consider in such an examination of knowledge about help-seeking is that there might be a range of culturally-sanctioned responses found to be effective for dealing with depression in this cultural context. Tribe [21] identifies the presence of ‘health pluralism’ in this system, where there is a multi-layered range of explanatory beliefs, help-seeking behaviours and a diverse group of designated helpers and healers. The use and endorsement of traditional practices such as approaching alternative medicine practitioners and Ayurvedic physicians or engaging in religious rituals, exorcism, tying of a charmed thread, prayer, blessings, holy gaze, sprinkling or application of holy materials, elimination of evil objects and horoscope reading have been observed among the Sri Lankan population when dealing with mental health problems [13, 22–25]. As seen in the mental health literacy survey of personal carers of the mentally ill [13] and other studies [23], such beliefs and practices of the population exist alongside their use and endorsement of getting help from professionals, such as psychiatrists or doctors, aligning with the notion of health pluralism. The prior mental health literacy surveys also found that participants endorsed informal help, such as from family [13, 14]. Hence, an assessment of depression literacy of undergraduates must also examine their beliefs about this wide variety of help-seeking options.

### Expert opinion as a benchmark for assessing mental health literacy

Knowledge of evidence-based practices underlies the notion of mental health literacy [12] and therefore, an evaluation of undergraduates’ knowledge about help-seeking must be viewed in light of such practices. However, given that culture can have an impact on the help-seeking beliefs and practices of undergraduates [3, 26], such an evaluation must also include these cultural elements, which might not be evidence-based, but contribute to the healing process, such as through placebo effects [12]. Both these aspects would be taken into account at least to some extent, when examining the depression literacy of a target population in reference to the related beliefs of mental health experts from the specific cultural context, who are knowledgeable about treatments routinely prescribed by professionals, but who are also knowledgeable of practices that have beneficial effects in their culture. Such methodology of assessing a population’s mental health literacy relating to recognition and treatment of disorders by using the corresponding responses of health professionals as the benchmark for comparison is analogous to criterion-referenced testing, and has been used previously [27–29].

This study aimed to examine the depression literacy of undergraduates in Sri Lanka focussing on problem-recognition, knowledge about dealing with depression, and the association between these aspects. Studies assessing recognition of depression among undergraduates have examined their ability to recognise the symptoms of depression or their ability to accurately label the condition when presented in a vignette [3, 4, 26, 30, 31]. Many studies have examined undergraduates’ knowledge about dealing with mental disorders by examining their personal help-seeking *intentions* if affected by the problem, and their *treatment beliefs* assessed by examining their perceptions about the helpfulness of different options of help [4, 32–35]. While both these aspects reflect knowledge about dealing with the examined disorders, they are also found to predict actual help-seeking behaviours [36]. Therefore, we attempted to answer the following research questions: (1) How would undergraduates label a vignette of depression? (2) What would undergraduates do if personally affected by depression? (3) What are the depression treatment beliefs of undergraduates (focussing on help-seeking perceptions) and how do these compare to expert opinion about dealing with depression? (4) Are the labels that undergraduates use for the vignette associated with their help-seeking intentions and treatment beliefs?

## Method

### Survey among undergraduates

#### Design, participants and setting

This cross-sectional study was conducted from June to November 2013 at the University of Colombo, one of the largest state universities in Sri Lanka [37]. Amarasuriya et al. [18] have previously reported data from this study on the prevalence of depression among undergraduates. The study was among undergraduates in all years of study at five of the six undergraduate faculties of the University of Colombo, namely the Faculties of Arts, Law, Management and Finance, Medicine and Science as well as the University of Colombo School of Computing. Second and third year students of the Faculty of Education who attend lectures at the Faculty of Arts were also approached during data collection at the latter faculty.

#### Measure

Mental health literacy surveys used among the adult population [10], as well as with undergraduates [4], provided the basic template for developing the current questionnaire. The questionnaire underwent several stages of adaptation including incorporation of items relevant to the target population and the broader Sri Lankan mental health context. Mental health literacy surveys previously used in Sri Lanka were also reviewed within this process [13, 14]. This measure was reviewed for cultural relevance by two panels, namely, mental health professionals in Sri Lanka and Sri Lankan postgraduates at the University of Melbourne who had completed their undergraduate studies in Sri Lanka. The adapted questionnaire was then translated from English into Sinhala and Tamil by two professional translators. The questionnaire was in two versions, as either English–Sinhala or English–Tamil, with both versions containing the questions in English and participants able to use the version with their preferred translation. Each version was checked for translation accuracy; the English–Sinhala version by a clinical psychologist, senior registrar and registrar in psychiatry, and the English–Tamil version by a clinical psychologist, all conversant in the translation languages. The questionnaire was piloted among ten undergraduates at the University of Colombo prior to finalisation. The questionnaire was titled “Problems faced by undergraduates” (see Additional file 1 for the English–Sinhala version of the questionnaire). Following is a description of its components relevant to this study.

The questionnaire consisted of a vignette of an undergraduate named “Z”, with Major Depression as per DSM-IV diagnostic criteria. Participants were asked to imagine that this person was of their own age and gender.

The vignette was as follows:

‘Z’ has been feeling unusually sad and miserable for the last few weeks. Even though ‘Z’ feels tired all the time ‘Z’ has difficulty falling asleep almost every night. ‘Z’ doesn’t feel like eating and has lost weight. ‘Z’ finds it difficult to concentrate on studies and ‘Z’s marks have dropped. ‘Z’ complains of feeling lifeless and finds even day to day tasks too much to handle. ‘Z’ finds it difficult to make decisions even about minor matters. ‘Z’ doesn’t want to go to university and tries to stay alone all the time. ‘Z’ seems very different to what ‘Z’ was like before. ‘Z’s’ parents and friends are very worried about ‘Z’.

This was followed by open-ended questions asking what was wrong with ‘Z’ (problem- recognition), what respondents would do if they had this problem (help-seeking intentions), and questions examining their perceptions about the helpfulness of a range of help-providers and interventions to help ‘Z’ to deal with the problem (rated as ‘very helpful’, ‘fairly helpful’, ‘neither helpful nor unhelpful’, ‘fairly unhelpful’, ‘very unhelpful’ and ‘don’t know’). The questionnaire also included the Patient Health Questionnaire-9, including its Sinhala/Tamil adaptations, validated for the Sri Lankan context [14]. Another study found the PHQ-9 to have good reliability and the categorical algorithm for Major Depression to have high specificity but relatively lower sensitivity, with the latter possibly due to the stringency of criteria used for diagnosis [38].

#### Procedure

The questionnaire was distributed, completed and returned during lectures common to each year of study at each of the identified Faculties/Schools. However, this was not possible at the Faculty of Arts due to the varied subject combinations within the Faculty. Therefore, lectures with the largest student cohorts were approached. During distribution of the questionnaires, the potential participants were given a brief introduction to the study, mostly by SDA (first author) or, in her absence, by the relevant lecturer who read out an introductory statement. The students were also informed that their identity would be anonymous in the study and that participation was voluntary. They were then referred to the participant information sheet. This was distributed with the questionnaires and provided more details about the study, including that if an answered survey was returned, this implied the respondent’s consent to participate in the study. The participants took approximately 20 min to complete the questionnaire.

##### Coding of open-ended questions

Coding was done by SDA, a clinical psychologist trained in Sri Lanka, who is fluent in Sinhala and English, the languages used by most participants. SDA coded the English translations of the Tamil responses, which were provided by a professional translator. Pre-coded categories used in similar research were used as a guideline when coding responses for the two questions [10, 39]. However, as this is the first study of such a nature among this undergraduate population, coding categories were created for all responses which varied in meaning, allowing the data to guide the creation of coding categories. For the problem-recognition question, identification of Sinhala words relevant to ‘depression’, which were ‘*visaadaya*’, ‘*maanasika avapeedanaya*’ and ‘*avapaathaya*’, was guided by words identified in previous depression-related publications [16]. Other labels given by participants that differed in meaning were coded as separate categories.

Each of the categories obtained for the two questions was coded as ‘yes’ or ‘no’ where multiple categories could be coded. Subsequent to this, the authors re-grouped the common coding categories, with the final categories being those nominated by ≥5 % of the respondents. However, in the case of the problem-recognition question, when a mental health-related label was nominated by ≥2 to ≤5 %  (the lower limit being approximately 90 responses), and indicated a distinct category approximating correct recognition of the condition, these were also permitted to constitute separate categories.

### Survey among mental health experts

#### Participants

Psychiatrists and clinical psychologists were identified through their respective professional/registration bodies, being 79 [40] and 23 (excluding SDA) (personal communication, Sri Lanka Medical Council, 31st October, 2013), respectively. A total of 66 psychiatrists and 19 clinical psychologists were contacted for the study.

#### Measure

This questionnaire consisted of extracts of the questionnaire used among the undergraduates, including the vignette of depression (where the condition was identified) and the questions assessing perceptions about the helpfulness of different options of help for depression. The items and rating scales used were the same as those used among the undergraduates.

#### Procedure

The questionnaire was administered online, with a link to it emailed to participants for whom email addresses could be obtained.

### Ethics approval

Approval was obtained from the Ethics Review Committees of the Faculty of Medicine, University of Colombo, and University of Melbourne.

### Statistical analysis

Descriptive data were analysed using valid percent frequencies and 95 % confidence intervals. Chi square tests were used to identify differences in problem-recognition in relation to the language used.

Separate binary logistic regression analysis models were used to examine if the label categories used in problem-recognition (IV) predicted undergraduates’ help-seeking intentions and treatment beliefs (DVs), while controlling for gender, faculty, year of study, age category, residence, religion, the presence of Major Depression as per the PHQ-9 [41], and language of response.

## Results

### Participant characteristics

#### Survey among undergraduates

Approximately 100 % of questionnaires distributed were returned. From these, a questionnaire was considered as valid if, apart from the demographic information questions, any item within the questionnaire was answered. This led to a total of 4671 questionnaire responses (exclusion of three questionnaires), with 96.0 % in the English–Sinhala version and the rest in the English–Tamil version. Table 1 presents the demographic characteristics of the sample, which was approximately 52 % of the undergraduates at the University of Colombo.

**Table 1:** Demographic and other characteristics of the undergraduates (n = 4671) and mental health experts (n = 37)

Variables	n	%
Undergraduates		
Gender		
Male	1447	31.0
Female	3220	68.9
Faculty		
Medicine	620	13.3
Arts and Education^a^	1198	25.6
Law	616	13.2
Management and finance	1025	21.9
Science	687	14.7
School of computing	524	11.2
Year of study		
1st year	1946	41.7
2nd year	1243	26.6
3rd year	838	17.9
4th year	530	11.3
5th year (Medicine)	114	2.4
Age group (Mean = 22.17; SD = 1.46)		
18–20 years	515	11.0
21–23 years	3355	71.8
24 and above	793	17.0
Ethnicity		
Sinhala	4281	91.7
Tamil	193	4.1
Sri Lankan moor	147	3.1
Other	46	1.0
Religion		
Buddhist	4064	87.0
Hindu	161	3.4
Islamic	152	3.3
Roman catholic	215	4.6
Other	73	1.6
Residence when going to University		
Home	1752	37.5
Hostel	1403	30.0
Rented place	1188	25.4
Home of friend or relative	272	5.8
Other	51	1.1
Screening positive for major depression (n = 4304)		
No	3903	90.7
Yes	401	9.3
Mental health experts		
Gender		
Male	9	62.2
Female	23	24.3
Age group		
Below 30 years	2	5.4
30–39 years	10	27.0
40–49 years	13	35.1
50–59 years	4	10.8
Above 60 years	4	10.8

^a^Those in the Faculty of Education were 5.6 % of this group

#### Survey among mental health experts

Responses of 37 experts, who were 36 % of the total number of mental health experts in the population, were obtained (Psychiatrists = 21; Psychologists = 12; not specified = 4). Table 1 presents demographic characteristics of the sample.

### Problem-recognition among undergraduates

From those who responded to this question (n = 4535), 41.2 % responded in English, 55.5 % in Sinhala, 2.1 % in Tamil, with the rest using more than one language. Table 2 shows the seven categories that emerged from the responses. The category ‘mental issue’ was used to collectively represent the range of labels relating to coding categories such as ‘*mental problem*’, ‘*mental unrest*’, ‘*being mentally in a mess*’, ‘*mental break down*’, as they had no clear distinction in meaning between one another. The label ‘mental illness’ constituted a separate category although nominated by ≤5 %, as it was distinct from the other label categories and approximated correct recognition of ‘depression’. All responses not relevant to the seven categories were included in an ‘other’ category. Although emotion-related labels were used by 9.3 %, these were disparate, with some reflecting the words provided in the vignette. Hence, these responses were also assigned to the ‘other’ category.

**Table 2:** Problem-recognition among undergraduates in relation to different label categories

Label category	Recognition percentage (95 % CI) (n = 4535)	Language of response					
		English		Sinhala		Tamil	
		% Sample (n = 4535)	% Language group (n = 1871–1880)	% Sample (n = 4535)	% Language group (n = 2521–2529)	% Sample (n = 4535)	% Language group (n = 93–95)
Depression	17.4 (16.3–18.5)	15.2	36.8	1.9	3.5	0.1	4.3
Mental illness	2.2 (1.8–2.7)	0.7	1.8	1.5	2.6	0	2.2
Mental issue	19.6 (18.5–20.8)	4.1	10.0	14.6	26.3	0.9	41.9
Stress/pressure/mental suffering	36.2 (34.8–37.6)	12.5	30.2	23.1	41.5	0.3	15.1
University/education related problems	23.7 (22.4–24.9)	9.6	23.2	13.3	23.9	0.6	27.7
Romantic relationship related problems	8.3 (7.5–9.1)	3.7	9.0	4.4	8.0	0.1	5.4
Other	29.3 (28.0–30.6)	10.9	26.5	17.4	31.2	0.8	40.0

Only a small proportion of responses were in more than one language. These results are not presented

% sample indicates use of the label categories in relation to the total sample

% language group indicates use of the label categories in relation to those responding within the particular language group

From the responses, 67.7 % were relevant to one category, with others relevant to two or more. Overall, the most common category nominated was ‘stress/pressure/mental suffering’ followed by ‘university/education problems’, with ‘depression’ and ‘mental illness’ nominated by fewer respondents. Although ‘depression’ was identified by 17.4 % of the sample, this recognition rate dropped to 10.8 % (n = 3939) when the Faculty of Medicine was excluded from the analysis. However, ‘depression’ was the most common category used by English-respondents. Also, 71.2 % of undergraduates used labels within the sphere of a mental health-related problem (‘depression’, ‘mental illness’, ‘mental issue’, ‘stress/pressure/mental suffering’).

Chi square tests examined if there were differences in the use of the label categories relating to the language of response. Due to the low response rate in Tamil, this analysis was only done for English and Sinhala responses. The category ‘depression’ was used significantly more in English, χ^2^(1, *N* = 778) = 679.63, *p* < 0.001, while the following categories were used significantly more in Sinhala: ‘mental issue’, χ^2^(1, *N* = 852) = 146.92, *p* < 0.001, ‘stress/pressure/mental suffering’ χ^2^(1, *N* = 1615) = 37.07, *p* < 0.001 and ‘other’, χ^2^(1, *N* = 1286) = 8.54, *p* = 0.003.

### Knowledge about dealing with depression among undergraduates

#### Intentions to seek help

Table 3 shows the undergraduates’ intended help-seeking actions if personally affected by the problem, with help from friends and parents being the most highly endorsed.

**Table 3:** Undergraduates’ help-seeking intentions if personally affected by problem (nominated by approximately ≥5 % of respondents)

Intended help-seeking actions	% Intending to seek help (95% CIs) (n = 4461)	
Professional/formal options		
Help from psychiatrist/related help	7.3	(6.6–8.0)
Help from doctor/getting medical treatment^a^	7.7	(6.9–8.4)
Help from counsellor/getting counselling	6.5	(5.7–7.2)
Informal options		
Help from friend	34.2	(32.8–35.6)
Help from parent	20.2	(19.0–21.4)
Help from family member/s	4.8	(4.2–5.4)
Help from person not specified	13.9	(12.9–14.9)
Doing enjoyable/relaxing/physical activities or taking a break	16.1	(15.0–17.2)
Dealing with educational difficulties/focussing on education/managing work	9.0	(8.1–9.8)
Using religious-oriented strategies	7.2	(6.4–7.9)
Understanding problem/self-initiated effort to get rid of problem	6.5	(5.7–7.2)

^a^The use of medication was coded in this category

#### Treatment beliefs

Options rated as either ‘helpful’ (‘very helpful’ or ‘fairly helpful’) or ‘unhelpful’ (‘very unhelpful’ or ‘fairly unhelpful’) by ≥75 % of the experts were established as the benchmarks for assessing the depression literacy of the undergraduates. Table 4 presents the proportion of undergraduates who rated each of these items as ‘helpful’ (‘very helpful’ or ‘fairly helpful’) or ‘unhelpful’ (‘very unhelpful’ or ‘fairly unhelpful’). Effect size estimates provided by Rosenthal [42] were used to estimate the differences between the ratings of the undergraduates and experts (i.e., the differences in the proportion of undergraduates and experts rating the options as ‘helpful’/‘unhelpful’), with differences of ≥18 and ≥30 indicating at least a medium and large effect size respectively (Tables 4). Options that ≥75 % of the undergraduates endorsed, but for which their rating deviated from expert opinion with a medium or large effect size, are also presented.

**Table 4:** Undergraduates’ treatment beliefs using expert opinion as a benchmark for comparison

Help-provider/intervention	Undergraduates (N = 4502–4651)		Mental health experts (N = 28–37)	
	Option rated as ‘Helpful’	Option rated as ‘Unhelpful’	Option rated as ‘Helpful’	Option rated as ‘Unhelpful’
	% (95 % CI)	% (95 % CI)	% (95 % CI)	% (95 % CI)
Professional/formal options recommended by experts				
Psychiatrist	88.9 (88.0–89.8)	2.4 (2.0–2.9)	100 (100–100)	0 (0–0)
Psychologist	66.7^a^ (65.3–68.0)	7.0 (6.3–7.7)	100 (100–100)	0 (0–0)
Counsellor	90.7 (89.9–91.5)	1.8 (1.4–2.2)	89.2 (78.7–99.7)	2.7 (0–8.2)
University student counsellor	75.3 (74.1–76.6)	5.3 (4.7–6.0)	75.0 (60.1–89.9)	2.8 (0–8.4)
Mental health professional at University Psychiatry Unit	70.5^b^ (69.2–71.8)	6.2 (5.5–6.9)	100.0 (100–100)	0 (0–0)
University Medical Officer	51.0^a^ (49.6–52.5)	11.3 (10.4–12.3)	88.9 (78.1–99.7)	2.8 (0–8.4)
Organisation helping people to deal with problems	48.7^a^ (47.3–50.2)	12.6 (11.6–13.5)	83.3 (70.5–96.1)	2.8 (0–8.4)
Get counselling or psychological therapy	88.5 (87.6–89.4)	2.2 (1.8–2.6)	100 (100–100)	0 (0–0)
Take western medicine to improve mood	29.0^a^ (27.7–30.3)	26.8^b^ (25.5–28.0)	97.1 (91.3–100)	0 (0–0)
Informal options recommended by experts				
Parents	93.0 (92.3–93.7)	1.6 (1.2–2.0)	78.4 (64.5–92.3)	10.8 (0.3–21.3)
Friend from University	91.6 (90.8–92.4)	1.3 (1.0–1.6)	88.6 (77.5–99.7)	2.9 (0–8.7)
Boyfriend/girlfriend/spouse	87.6 (86.7–88.6)	1.7 (1.3–2.1)	83.8 (71.3–96.2)	2.7 (0–8.2)
Do enjoyed activities	97.0 (96.5–97.5)	0.8 (0.5–1.0)	94.1 (85.8–100)	0 (0–0)
Meditation, yoga and relaxation exercises	93.5 (92.8–94.2)	1.8 (1.4–2.2)	80.0 (66.1–93.9)	5.7 (0–13.8)
Become more active	92.0 (91.3–92.8)	2.1 (1.7–2.5)	85.3 (72.8–97.8)	5.9 (0–14.2)
Physical exercise	85.6 (84.6–86.6)	2.7 (2.2–3.2)	82.9 (69.7–96.0)	0 (0–0)
Talk to others who have faced similar problems	76.5 (75.3–77.8)	7.2 (6.5–8.0)	85.7 (73.5–97.9)	5.7 (0–13.8)
Cut down use of alcohol/cigarettes/drugs	76.5 (75.3–77.8)	6.1 (5.4–6.7)	94.1 (85.8–100)	2.9 (0–8.9)
Improve sleeping habits	74.3 (73.1–75.6)	8.2 (7.4–9.0)	85.7 (73.5–97.9)	0 (0–0)
Get information from internet about dealing with problem	41.4^a^ (40.0–42.8)	19.3 (18.2–20.5)	80.0 (66.1–93.9)	0 (0–0)
Options identified as ‘unhelpful’ by experts				
Deal with problem alone	17.3 (16.2–18.4)	62.8^b^ (61.4–64.2)	0 (0–0)	86.1 (74.2–98.0)
Stop going to university	6.4 (5.7–7.1)	78.9 (77.7–80.1)	2.9 (0–8.7)	91.4 (81.7–100)
Use alcohol/cigarettes/drugs	1.8 (1.4–2.1)	90.2 (89.4–91.1)	0 (0–0)	94.3 (86.2–100)
Options not recommended by experts but rated as ‘helpful’ by ≥75 % of undergraduates				
Clergy/religious priest	81.1^b^ (80.0–82.3)	4.0 (3.4–4.6)	54.3 (36.9–71.6)	17.1 (4.0–30.3)
Perform religious activities	92.9^b^ (92.2–93.7)	1.6 (1.3–2.0)	63.6 (46.3–81.0)	6.1 (0–14.7)

^a^Where difference in rating between undergraduates and mental health experts is at least a large effect size (≥30 %)

^b^Where difference in rating between undergraduates and mental health experts is at least a medium effect size (≥18 %)

### Association between problem-recognition and knowledge about help-seeking (help-seeking intentions and treatment beliefs)

As the study attempted to examine the association between the use of different labels and knowledge about help-seeking, and as multiple responses were permitted for the problem-recognition question, all responses relevant to the four mental health-related label categories (‘depression’, ‘mental illness’, ‘stress/pressure/mental suffering’, ‘mental issue’) were recoded as per a hierarchy for this analysis. If participants used the label ‘depression’ as well as another label relevant to the other three label categories, responses were only coded for ‘depression’. If responses were relevant to the category ‘mental illness’ and ‘stress/pressure/mental suffering’ or ‘mental issue’, responses were only coded for ‘mental illness’. Given the range of mental health-related labels that emerged for the ‘mental issue’ category and inability to distinguish whether these labels or those relating to ‘stress/pressure/mental suffering’ approximated depression better, responses relevant to both these categories were coded if used together.

Tables 5 and 6 present the adjusted odds ratios obtained from the analyses examining the association between the label categories (predictor) and the undergraduates’ help-seeking intentions and perceptions relating to professional/formal help. When examining the overall trends, recognition of the problem as ‘depression’, ‘mental illness’, ‘mental issue’ or ‘stress/pressure/mental suffering’, seem to be associated with higher odds of intending to seek professional/formal help (Table 5), as well as higher odds of perceiving these options as being ‘helpful’ (Table 6). However, recognition of the problem as an education or romantic related issue seem to be associated with lower odds of intending to seek help from these options (Table 5) and lower odds of perceiving them as being ‘helpful’ (Table 6). Furthermore, label specificity or recognising the problem as ‘depression’, as compared to using other mental health-related labels, seems to be associated with higher odds of endorsing a wider range of both medically and psychologically oriented help-seeking options (all professional/formal options in Table 5 and six of the nine options in Table 6). Labelling the problem as a ‘mental illness’ also seems to be associated with higher odds of endorsing many of these professional/formal options. However, the results also show to some extent that labelling the problem as a ‘mental illness’ or ‘mental issue’ is associated with higher odds of endorsing more medical-oriented options, while labelling it as ‘stress, pressure, mental suffering’ is associated with higher odds of endorsing more psychologically-oriented options. In most instances that a mental health-related label was used, regardless of the type of label, the odds of endorsing psychiatrists were higher.

**Table 5:** Examination of label categories used in problem-recognition as predictors of intending to seek professional/formal help (n = 4081)

Intended help-seeking actions	Label category					
	Depression	Mental illness	Mental issue	Stress/pressure/mental suffering	University/education related problem	Romantic relationship related problem
	(Adjusted odds ratio)	(Adjusted odds ratio)	(Adjusted odds ratio)	(Adjusted odds ratio)	(Adjusted odds ratio)	(Adjusted odds ratio)
Help from psychiatrist/related help	4.26***	4.74***	1.50**	0.79	0.28***	0.25**
	(2.96–6.14)	(2.84–7.91)	(1.13–2.00)	(0.61–1.04)	(0.18–0.43)	(0.11–0.56)
Help from doctor/getting medical treatment	1.46*	2.76***	1.73***	0.93	0.45***	0.18***
	(1.04–2.06)	(1.58–4.84)	(1.31–2.27)	(0.72–1.19)	(0.32–0.63)	(0.08–0.42)
Help from counsellor/counselling	1.88***	1.19	0.93	1.27	0.53**	0.50*
	(1.32–2.69)	(0.51–2.78)	(0.66–1.31)	(0.97–1.66)	(0.37–0.76)	(0.27–0.94)

* *p* < 0.05; ** *p* < 0.01; *** *p* < 0.001

**Table 6:** Examination of label categories used in problem-recognition as predictors of rating recommended professional/formal option as ‘helpful’ (n = (4042–4149)

Help providers/interventions	Label category					
	Depression	Mental illness	Mental issue	Stress/pressure/mental suffering	University/education related problem	Romantic relationship related problem
	(Adjusted odds ratio)	(Adjusted odds ratio)	(Adjusted odds ratio)	(Adjusted odds ratio)	(Adjusted odds ratio)	(Adjusted odds ratio)
Psychiatrist	1.72**	5.16^*^	1.51**	1.35*	0.52***	0.46***
	(1.23–2.39)	(1.26–21.20)	(1.12–2.05)	(1.07–1.70)	(0.42–0.65)	(0.34–0.61)
Psychologist	1.36**	2.19**	1.11	1.05	0.76**	0.57***
	(1.08–1.71)	(1.28–3.75)	(0.93–1.32)	(0.91–1.21)	(0.65–0.90)	(0.45–0.72)
Counsellor	0.98	1.14	1.09	1.39**	1.09	0.56**
	(0.69–1.39)	(0.52–2.52)	(0.80–1.47)	(1.08–1.78)	(0.84–1.42)	(0.41–0.78)
University student counsellor	1.27*	1.06	1.11	1.17	1.18	0.76*
	(1.01–1.61)	(0.63–1.80)	(0.91–1.35)	(0.99–1.37)	(0.99–1.41)	(0.59–0.98)
Mental health professional at University Psychiatry Unit	1.27*	2.00*	1.21	1.16	0.71***	0.82
	(1.02–1.57)	(1.12–3.59)	(1.00–1.46)	(1.00–1.35)	(0.61–0.84)	(0.65–1.05)
University Medical Officer	1.11	1.89**	1.20*	1.07	0.82^*^	0.69**
	(0.91–1.35)	(1.18–3.01)	(1.02–1.42)	(0.94–1.23)	(0.71–0.96)	(0.54–0.87)
Organisation helping people to deal with problems	1.16	1.49	1.01	1.00	1.00	0.80
	(0.95–1.41)	(0.96–2.30)	(0.86–1.19)	(0.87–1.14)	(0.86–1.16)	(0.63–1.00)
Get counselling or psychological therapy	1.67**	2.57	1.17	1.31*	0.75*	0.59***
	(1.20–2.33)	(0.93–7.08)	(0.89–1.54)	(1.05–1.64)	(0.60–0.94)	(0.43–0.79)
Western medicine to improve mood	1.46***	2.26***	1.25*	1.02	0.66***	0.62**
	(1.18–1.79)	(1.46–3.51)	(1.04–1.49)	(0.88–1.18)	(0.56–0.79)	(0.47–0.83)

* *p* < 0.05; ** *p* < 0.01; *** *p* < 0.001

## Discussion

This paper examines depression literacy of University of Colombo undergraduates in the areas of problem-recognition, help-seeking intentions and treatment beliefs, and the associations among these. Although a majority of undergraduates recognised the problem as a mental health problem, only 17.4 % recognised it as depression; this rate being lower if those from the Faculty of Medicine were excluded. Their perceptions about the helpfulness of professional help-providers, such as psychiatrists and counsellors, and interventions, such as counselling/psychological therapy, align with expert opinion. However, parents and friends were the most highly endorsed help-providers by the undergraduates, with more undergraduates indicating that they would seek help from them, than from professionals when personally dealing with the problem. Furthermore, only a low percentage rated ‘western medicines to improve mood’ as ‘helpful’, deviating considerably from expert opinion. In contrast, undergraduates highly endorsed religious-oriented help and strategies, as well as self-help strategies, such as doing enjoyable activities, and indicated their intentions to use these if affected by the problem. Their ability to identify the problem as being mental health-related was associated with their higher literacy of professional/formal help. Furthermore, label specificity with regard to the use of the label ‘depression’ was associated with their endorsement of more professional/formal options of help, as well as a wider range of both medically and psychologically oriented options that were recommended by experts. Following is a more in-depth discussion of the findings.

### Problem-recognition

Interpretation of the low rate of recognition of depression must be seen in light of the word ‘depression’ not being in the common lay Sri Lankan vocabulary, especially in the case of Sinhala words for depression. Although there was low use of labels relating to this category among the Sinhala-respondents, ‘depression’ was the most predominantly used label by those responding in English. Furthermore, while use of the ‘depression’ label category was significantly higher among English-respondents, labels relating to the categories ‘mental issue’ and ‘stress/pressure/mental suffering’ were used significantly more by Sinhala-respondents, with the latter category used by over 40 % of those in this language group. Hence, the rate of recognition of depression among the Sinhala-group could either be due to their *actual* low level of depression literacy, where they recognised the problem as one of a lesser severity such as ‘stress’, or alternatively could be due to recognition of the problem being intertwined with linguistics where, in the absence of lay terms for depression, they used more easily accessible terms such as ‘stress’ (*maanasika aathathiya*/*maanasika peedanaya*) or those relevant to the ‘mental issue’ category. Interestingly, this latter label category encompassed a range of words in Sinhala, relating to *mental problem*, *mental unrest*, *mentally in a mess*, *mental break down*, and could have been the respondents’ attempts to capture the state of depression using these terms, in the absence of a label for depression within their language domain. The wide array of words used to label the condition and the varying severity of these from a mental health point of view (stress vs. depression) makes it difficult to distinguish the respondents’ perception of the problem and where they would position it on the mental health spectrum, i.e. if they label the condition as *mental unrest*, would this warrant professional help or is it the type of *mental unrest* they experience due to a relationship breakup where they might choose to deal with it alone?

Even if only considering the rate of recognition among those responding in English, this is lower than recognition rates found among undergraduates in countries such as Australia [4]. Although this rate is more comparable to those found in other non-western contexts in which, as in the present study, participants were not native English speakers [43, 44], it would be expected that recognition of depression would have been higher among the present sample consisting of undergraduates. Hence, the findings indicate the need to improve this population’s ability to recognise the condition.

Over 20 % of undergraduates identified the problem as education-related. Although respondents have attributed causal factors to the case instead of labelling the problem, the proportion of students identifying their university experience and education-related activities as the cause for the described symptoms of depression, calls for the urgent attention of university educators.

### Help-seeking intentions and treatment beliefs

The low endorsement of “western medicine to improve mood” and high endorsement of counselling/psychological therapy corroborate previous findings from both eastern and western contexts of undergraduates perceiving antidepressants as less helpful, and showing greater preference towards psychologically-oriented interventions [4, 45]. However, we also found that in addition to their low ‘helpful’ rating, a quarter of undergraduates rated these medications as ‘unhelpful’. Hence, such beliefs of undergraduates in Sri Lanka must be addressed, as they might act as potential barriers when treating those who are depressed with antidepressant medication.

We also found that the undergraduates gave a relatively low ‘helpful’ rating for psychologists, deviating from expert opinion. However, these findings contradict their high ‘helpful’ rating for counselling and psychological therapies, which are within the purview of a psychologist’s service provision. These findings might be due to the undergraduates’ lack of awareness about the role of psychologists given the low number of clinically specialised psychologists in the country and their minimal exposure to this professional category.

Although the undergraduates acknowledged the helpfulness of psychiatrists and counsellors when dealing with the problem, their endorsement of student counsellors, mental health professionals and medical officers within the university system was lower, with their ‘helpful’ ratings for the latter two groups deviating from expert opinion. This is concerning, as these individuals are some of the key persons available to assist distressed undergraduates. At present, academic or other university staff appointed to the role of university counsellors and the university medical officer act as contact points for undergraduates requiring help. They refer students needing mental health assistance to mental health professionals who are staff or affiliated to the university. Although reluctance of students to seek support from universities is generally observed [46], this issue must be especially addressed in countries such as Sri Lanka where mental health resources are limited and where the establishment of effective and feasible mental health services might rely heavily on existing support services.

Our findings that undergraduates highly endorse informal sources, such as parents and friends, have also been observed cross-culturally [3], indicating their importance in the healing process of depressed undergraduates. These findings are of greater significance in countries like Sri Lanka, which have an inadequate number of mental health professionals and a lack of specialised mental health services in universities, where help-providers such as parents and friends might be the first points of contact for undergraduates. This highlights the need to provide appropriate mental health first aid training [47] to these social networks to assist depressed undergraduates.

Other informal help-providers that might need to be educated about providing assistance to distressed undergraduates are the clergy. Although these help-providers and religious strategies were not endorsed as being ‘helpful’ by the Sri Lankan mental health experts, whose opinions aligned with international expert consensus [29], the high endorsement of such help-providers and practices by undergraduates indicates that they perceive such help as being integral to the healing process. Hence, it is necessary to involve these help-providers as partners in initiatives aiming to improve the depression literacy of undergraduates, to ensure that they seek and adhere to appropriate treatments [12]. The undergraduates’ endorsement of such practices alongside professional help also indicates the existence of health pluralism in their help-seeking.

A high proportion of undergraduates endorsed evidence-based self-help activities, such as doing enjoyable activities, becoming more active and physical exercise [48, 49], and indicated their intentions of engaging in relaxing/enjoyable activities when personally dealing with the problem. The findings indicate the potential use of these strategies in the behavioural activation of depressed undergraduates. Although there is comparatively less evidence for strategies such as relaxation exercises, yoga and meditation [48–50], these were highly endorsed by both the undergraduates and mental health experts. Indian undergraduates have also endorsed such methods, with their endorsement of these being higher than endorsements by American undergraduates [51]. Hence, the effectiveness of these strategies needs to be examined in cultures with Indo-Aryan influences such as Sri Lanka, as these might be linked to the health belief systems of these populations [52, 53]. However, given that the undergraduates’ ‘unhelpful’ rating for the option of *dealing with the problem alone* deviated from expert opinion, it is necessary to ensure that undergraduates do not resort to only using such self-help strategies for depression and that they are educated on the need to use these complementary to other professional help.

### Association between problem-recognition and knowledge about help-seeking

The findings suggest that the use of the label ‘depression’ is associated with better literacy about treatment options, aligning with previous research [19, 20]. This highlights the lack of lay words for depression in the Sinhalese vocabulary, and findings of the low recognition rate of depression among undergraduates, as being problematic. The absence of a unifying concept or lay term for depression could also pose challenges for depression literacy initiatives, making educational efforts relating to the topic difficult. Hence, it would seem that the introduction of depression-related labels into the lay vocabulary is necessary. However, this would require a systemic change over an extended period. An intermediate goal might be to improve the population’s ability to recognise the problem as being a mental health-related problem, as the findings indicate that this might also trigger some of the recommended help-seeking actions. However, this needs to be done with caution given our findings of the wide array of mental health-related words used by the study population to label the condition and the varying interpretations these might lead to. Hence, it might be more suitable to help the population to at least recognise the problem as a ‘mental illness’, as this label was also associated with endorsement of many of the professional/formal options. However, this label category was the least used by the study population (2.2 %).

The limitations of these findings in predicting actual help-seeking behaviours of undergraduates must be considered [4, 54]. Although the study measure was culturally adapted, with expert opinion used as the validity standard for assessing depression treatment beliefs of the sample, future work to examine its psychometric properties in this cultural context is needed. Holistically the vignette reflects someone affected by depression. However, the different symptoms presented are also relevant to some anxiety disorders and might have led participants to misclassify the problem. Also, the methodology considered an accurate label of ‘depression’ as being synonymous with the ability to recognise the condition. However, participants might have been able to accurately recognise the symptoms of depression, but been limited in their ability to express their understanding of the problem as a construct. The use of qualitative methodology would facilitate a more in-depth analysis of such aspects of problem-recognition and also help to further explore the help-seeking knowledge of the population.

## Conclusions

The low rate of problem-recognition found in this undergraduate population must be addressed. However, the findings must be considered in light of the lack of lay terminology for the condition. Nevertheless, label specificity, in this case, recognition of depression, is associated with better knowledge of help-seeking with regard to endorsing a wider range of professional help-seeking options, further emphasising the importance of addressing the low rate of depression recognition. Given the lack of lay terminology for depression, it is suggested that as an intermediate goal, depression literacy initiatives improve the population’s ability to recognise the problem as being mental health-related, and to use labels such as ‘mental illness’, as the findings indicate that this too might trigger some of the recommended help-seeking actions. Overall, the findings show that undergraduates are likely to consider the help of informal options such as parents and friends more than the help of professional/formal options, and that they might also seek religious help for their problems. Hence, depression literacy initiatives must improve the depression literacy of such informal or culturally relevant help-providers while also improving the undergraduates’ literacy of professional help, especially with regard to the use of medication for their depression.

## Additional file

10.1186/s13104-015-1589-7 English-Sinhala version of questionnaire.

## Abbreviations

DSM: diagnostic and statistical manual of mental disorders
PHQ-9: Patient Health Questionnaire-9
